# Hemodynamic alterations with large spontaneous splenorenal shunt ligation during adult deceased donor liver transplantation

**DOI:** 10.3389/fsurg.2022.916327

**Published:** 2022-10-17

**Authors:** Guangshun Chen, Qiang Li, Zhongqiang Zhang, Bin Xie, Jing Luo, Zhongzhou Si, Jiequn Li

**Affiliations:** ^1^ Department of Liver Transplant, The Second Xiangya Hospital of Central South University, Changsha, China; ^2^ Transplant Medical Research Center, The Second Xiangya Hospital of Central South University, Changsha, China

**Keywords:** portal blood flow volume, liver transplantation, hemodynamic consequences, spontaneous splenorenal shunts, portal vein thrombus

## Abstract

**Background:**

A large spontaneous splenorenal shunt (SRS) will greatly impact portal inflow to the graft during liver transplantation (LT). Direct ligation of a large SRS is an uncommon surgical procedure and the hemodynamic consequences of this procedure are unknown.

**Methods:**

In this retrospective study, we described our technique for direct ligation of a large SRS and the consequent hemodynamic changes during LT. 3-Dimensional computed tomography and Doppler ultrasonography were used to evaluate SRS and portal vein blood flow volume (PFV).

**Results:**

A total of 22 recipients had large SRS including 13 with PFV <85 ml/min/100 g (ligation group) and 9 with PFV ≥85 ml/min/100 g (no ligation group). The diameter of SRS was significantly larger in the ligation group than in the non-ligation group (22.92 ± 4.18 vs. 16.24 ± 3.60 mm; *p *= 0.0009). In all ligation patients, the SRS was easily identified and isolated, it was located just below the distal pancreas and beside the inferior mesenteric vein. PV flow increased significantly from 68.74 ± 8.77 to 116.80 ± 16.50 ml/min/100 g (*p *< 0.0001) after ligation; this was followed by a reduction in peak systolic velocity of the hepatic artery from 58.17 ± 14.87 to 46.67 ± 13.28 cm/s (*p *= 0.0013).

**Conclusions:**

Direct ligation of large SRS was an effective and safe surgical procedure to overcome the problem of portal hypoperfusion during LT.

## Introduction

Whole liver transplantation (LT) requires a hepatopetal portal inflow of at least 1000 ml/min to ensure proper liver function (1, 2). The portal vein (PV) flow to the liver graft is mainly determined by graft weight and quality, the patency of the portomesenteric venous system, diameter of the recipient's PV, and presence of portosystemic shunt (3, 4). A spontaneous splenorenal shunt (SRS) was found in 20%–35% of LT candidates (4–6). The presence of a large portosystemic shunt (>10 mm in diameter) will greatly impact portal inflow to the graft during LT (4, 6), potentially causing portal flow steal and leading to PV thrombus, hepatic encephalopathy, and graft dysfunction if the shunt is left without intervention (7–9).

Surgical procedures used for SRS during LT include renoportal anastomosis (RPA) (4, 10, 11), left renal vein ligation (LRVL) (4, 11, 12), splenectomy (13), and splenic vein ligation (11). There have been relatively few reports of direct SRS ligation (6) and these studies only focused on the surgical technique, with no details on the hemodynamic consequences of the procedure. In this retrospective study, we described our technique for direct ligation of a large SRS during LT and the consequent hemodynamic changes.

## Materials and methods

### Study protocol

Three-dimensional computed tomography (3D CT) and Doppler ultrasonography (US) were used to evaluate the SRS. A large SRS was defined as a shunt with diameter >10 mm before transplantation. Between January 1, 2017 and December 31, 2020, 720 adults (age ≥18 years) underwent deceased donor LT at the Second Xiangya Hospital of Central South University. A total of 22 recipients (3.05%) who were found to have a large SRS (including 1 patient with a large gastrorenal shunt [GRS]) were retrospectively analyzed. The study protocol conformed to the ethics guidelines of the 1975 Declaration of Helsinki and was approved by Institutional Review Board of the Second Xiangya Hospital of Central South University (No. 2019–050). Written informed consent was obtained from all participants. The transplantations were performed according to the Declaration of Istanbul, and no executed prisoners were used as donors.

### Intraoperative hepatic flow measurement

Intraoperative and postoperative PV blood flow volume (PFV) was evaluated by color Doppler ultrasound (US). 3D CT was routinely performed before and after LT to assess portosystemic shunts. The Doppler US parameters for blood flow features were calculated using the Logiq P5 US System (GE Healthcare, Little Chalfont, UK). PFV was calculated as cross-sectional area × portal blood velocity (angle-corrected) (14). Donor livers were weighed after back-table preparation. PFV was indexed to the graft weight (ml/min/100 g). Hepatic artery (HA) flow was determined based on the inner diameter of the HA, angle-corrected peak systolic velocity (PSV), end diastolic velocity (EDV), and resistive index (RI). To better evaluate the perfusion of the liver grafts and minimize the influence of graft weight on the measured flows, PFV (ml/min/100 g) in the non-SRS cohort 100 deceased adult liver transplant donors were presented before the study of SRS ligation was done. The median PFV was 124 ml/min/100 g (range, 46∼238 ml/min/100 g); only 10% of patients had a value <85 ml/min/100 g before skin closure. Based on these data and those from previous studies (1, 3), 85 ml/min/100 g was used as the PFV threshold for direct ligation of large SRS in this study.

### Surgical technique

**Figure 1:**
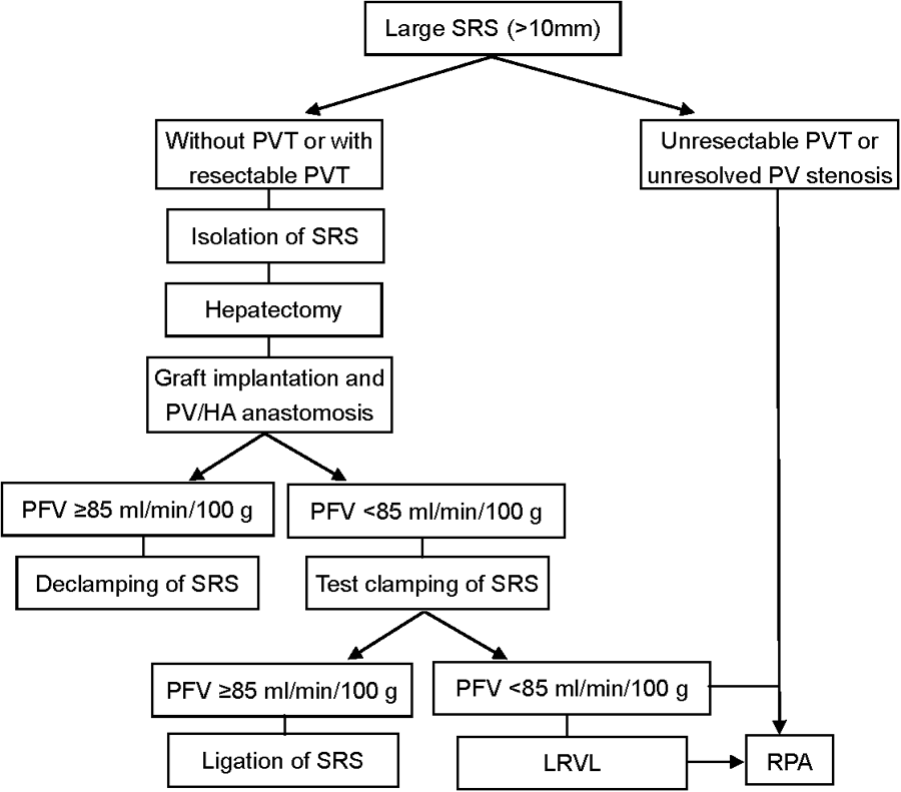
Flowchart for SRS ligation surgery selection.

The operations were performed by the same surgical team with the same classical orthotopic technique or piggyback methods. The flowchart for the surgical management of large SRS is shown in Figure 1. After removing the recipient's diseased liver, the SRS was identified and isolated as previously described (6). The SRS was located just below the distal pancreas and beside the inferior mesenteric vein. If there was PV stenosis or thrombosis, we performed PV thrombectomy and/or angioplasty. With the recipient liver removed, the donor liver was anastomosed to the appropriate structures for placement in an orthotopic position. The new liver was then allowed to reperfusion and the HA was anastomosed. We then evaluated PV and HA flow by Doppler US, with test clamping of the SRS if PFV was <85 ml/min/100 g. When there was a marked increase in PFV (≥85 ml/min/100 g), the SRS was ligated proximally to the juncture with the left renal vein (LRV) using nonabsorbable sutures.

### Liver biopsy evaluation and postoperative management

Liver biopsy routinely performed at the end of liver graft harvesting and LT. The same (blinded) pathologist evaluated all liver samples. Grafts were also evaluated for the presence of ischemia reperfusion injurys (IRI). IRI defined by the combination of apoptotic hepatocytes and inflammatory neutrophilic polynuclear's infiltrate throughout the lobule and around centrilobular veins. An IRI was graded as absent, mild, moderate, or severe. Severe IRI required centrocentrilobular necrosis (4). 3D CT was routinely performed on post-operative days (POD) 7 to assess the changes of collaterals and portal flow. Daily color Doppler US, biological tests and close clinical surveillance were conducted until POD 7, and then once every 3 days until discharge. After their discharge, all patients regularly visited our outpatient department: once a month for the first 6 months, and then once every 3 months.

### Data analysis

Values were expressed as mean ± SD. Statistical analysis of hemodynamic consequences were performed with the Student's t test. SPSS 11.0 (IBM Corp., USA) statistical software were used for statistical analyses and differences with *p *< 0.05 were considered statistically significant.

## Results

**Figure 2:**
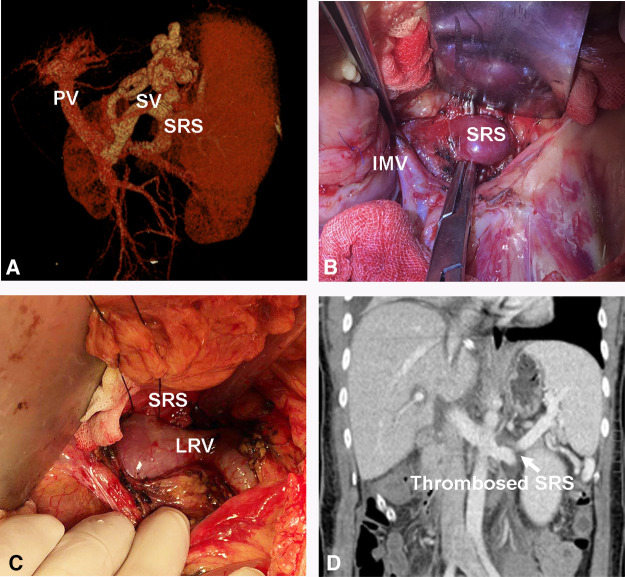
Direct ligation of a large SRS. (**A**) Large SRS before transplantation. (**B, C**) Isolation and ligation of large SRS. (**D**) Thrombosed SRS on day 7 post transplantation. SV, Splenic vein; IMV, inferior mesenteric vein.

**Figure 3:**
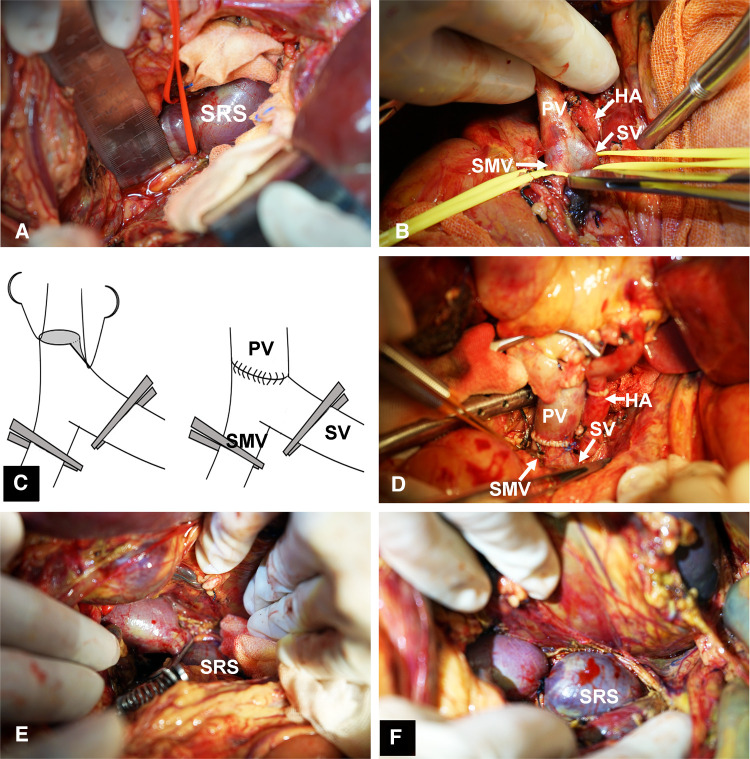
Surgical procedure for SRS ligation. (**A**) Identification and isolation of the large SRS. (**B**) Small diameter of PV,the isolation of SMV, and SV behind the neck of pancreas. (**C**) Angioplasty of the PV. (**D**) Portal vein anastomosis to the confluence of SV and SMV with satisfactory results. (**E**) Test clamping of SRS. (**F**) Ligation of SRS. SMV, superior mesenteric vein.

**Figure 4:**
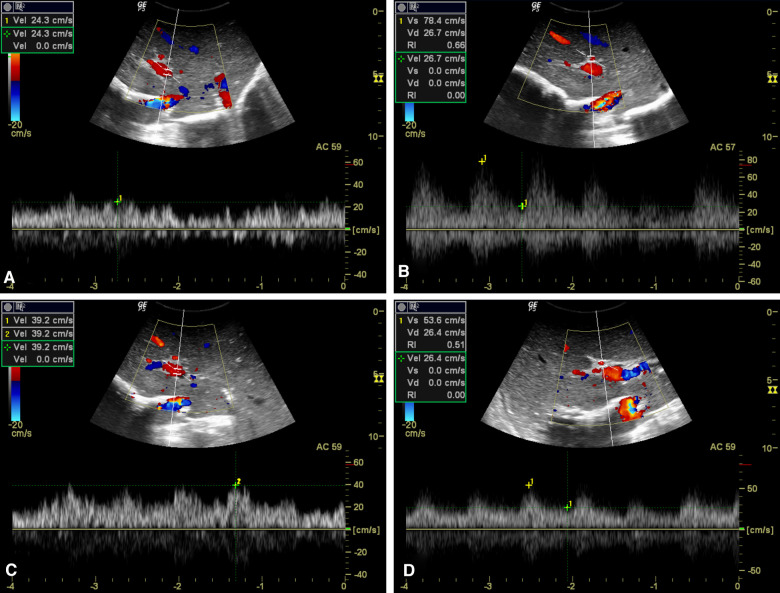
Hemodynamic consequences of test clamping of SRS. (**A, C**) PV flow increased markedly after test clamping of the SRS, from 24.3 cm/s (**A**) to 39.2 cm/s (**C**). (**B, D**) Decreased PSV of HA in response to SRS blocking from 78.4 cm/s (**B**) to 53.6 cm/s (**D**).

**Figure 5:**
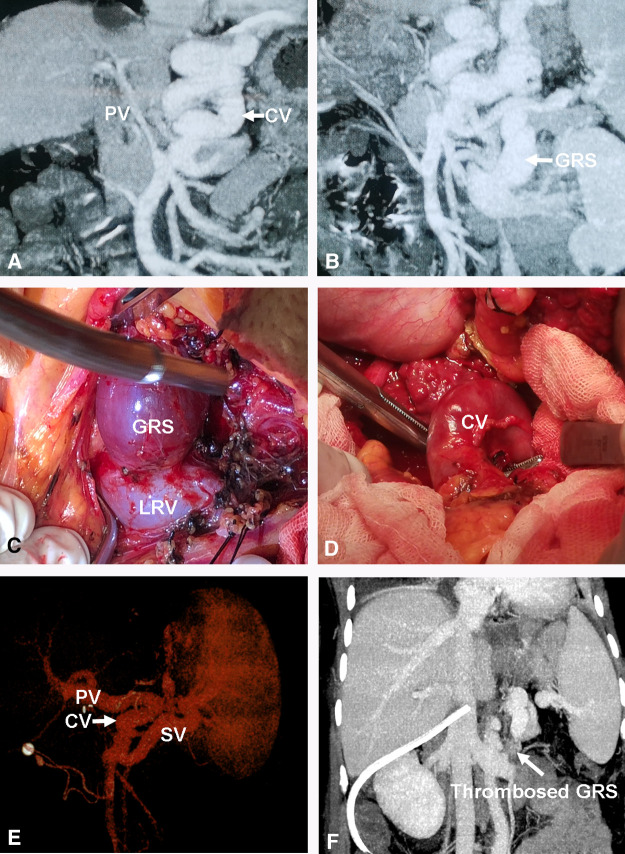
Direct ligation of a large GRS and reconstruction of gastric CV with PV. (**A**) Small diameter PV or cavernous transformation of PV. (**B**) Large GRS before transplantation. (**C**) Isolation of large GRS. (**D**) Isolation of gastric CV. (**E**) The images of the PV reconstruction with recipient gastric CV and graft PV on 3D CT. (**F**) Thrombosed GRS on day 7 post transplantation. CV, coronary vein; GRS, gastrorenal shunt.

The preoperative characteristics and flow measurements of cases with large SRS are shown in Tables 1, 2. To illustrate the SRS ligation procedure and the impact on PVF during LT, each figure corresponds to a case listed in Table 1 (ie, Figure 2 correspond to case no. 5, Figures 3, 4 correspond to case no. 13 and Figure 5 correspond to case no. 7). The SRS was located just below the distal pancreas and beside the inferior mesenteric vein (Figures 2B, 3A, 5C). They were easily identified and isolated, and no procedure-related complications were found in patients who underwent direct SRS ligation.

**Table 1:** Preoperative characteristics and flow measurements of liver transplant recipients with direct ligation of a large splenorenal shunt.

No.	Sex	Age (years)	PVT	Diagnosis	MELD score	SRS (mm)	PVD (mm)	PFV (B) (ml/min/100 g)	PFV (A) (ml/min/100 g)	PFV (7) (ml/min/100 g)	PSV (B) (cm/min)	PSV (A) (cm/min)	PSV (7) (cm/min)	EDV (B) (cm/min)	EDV (A) (cm/min)	EDV (7) (cmmin)	RI (B)	RI (A)	RI (7)
1	M	48	Partial	PBC	28	23.7	9.0	61.04	103.01	114.45	35	30	40	11	12	15	0.68	0.60	0.63
2	F	63	None	PBC + HCV	22	24.3	7.8	53.22	94.15	85.97	56	54	44	17	12	19	0.70	0.77	0.57
3	M	46	Partial	HBV + ALD	13	27.2	9.0	60.69	121.39	105.78	53	40	67	14	33	8	0.74	0.80	0.54
4	M	49	None	HBV	30	26.5	10.2	76.57	110.26	NA	41	35	NA	12	10	17	0.71	0.70	NA
5	M	42	None	HBV	27	17.6	10.3	66.13	101.41	92.88	45	32	36	20	11	12	0.56	0.65	0.67
6	F	52	Complete	AILD	19	25.8	9.0	81.22	120.61	110.76	70	76	90	25	26	22	0.64	0.65	0.75
7	F	57	CTPC	PBC	24	26.4 (GRS)	10.0	77.95	126.03	NA	47	41	NA	10	8	NA	0.79	0.80	NA
8	F	45	Partial	AILD	22	16.8	10.8	78.48	149.12	119.69	66	44	48	24	11	13	0.64	0.75	0.73
9	M	55	Partial	HBV + HCC	18	21.3	9.6	69.45	114.60	131.96	72	58	64	22	18	19	0.69	0.69	0.70
10	M	43	Partial	PBC + HBV	32	26.5	12.2	64.09	154.23	112.17	55	40	48	15	10	17	0.73	0.75	0.69
11	M	42	None	HBV	26	14.6	9.6	NA	142.62	110.07	NA	48	45	NA	10	12	NA	0.79	0.73
12	M	61	None	HBV + HCC	18	22.3	10.8	73.25	115.98	124.12	80	56	65	22	16	20	0.73	0.71	0.66
13	F	23	Partial	PFIC	22	25	9.8	62.81	102.88	112.70	78	54	61	27	26	19	0.66	0.51	0.69

(7), on day 7 post transplantation; (A), after ligation; (B), before ligation; F, female; M, male; AILD, autoimmune liver disease; ALD, alcohol-associated liver disease; CTPV, cavernous transformation of portal vein; HBV, hepatitis B virus related liver diseases; HCC, hepatocellular carcinoma; HCV, hepatitis C virus related liver diseases; MELD, model for end-stage liver disease; PBC, primary biliary cirrhosis, PVD, portal vein diameter.

**Table 2:** Preoperative characteristics and flow measurements of liver transplant recipients without ligation of large splenorenal shunt.

No	Sex	Age (years)	PVT	Diagnosis	MELD score	SRS (mm)	PVD (mm)	PFV (B) (ml/min/100 g)	PFV (7) (ml/min/100 g)	PSV (B) (cm/min)	PSV (7) (cm/min)	EDV (B) (cm/min)	EDV (7) (cm/min)	RI (B)	RI (7)
1	M	52	Partial	HBV	18	16.6	10.8	104.23	120.28	60	52	23	17	0.63	0.67
2	M	48	Partial	HBV	21	13.7	9.0	107.30	134.12	54	50	17	18	0.69	0.64
3	M	58	None	HBV + HCC	13	10.1	10.2	132.92	1 23.31	62	60	33	20	0.47	0.66
4	M	56	Partial	HBV + HCC	32	15.5	7.8	91.65	101.14	78	63	21	16	0.73	0.75
5	F	61	Partial	PBC	25	20.5	9.8	89.96	98.95	45	40	10	18	0.78	0.55
6	M	49	None	HBV	30	14.5	9.8	113.09	105.23	48	55	16	23	0.67	0.58
7	F	52	None	AILD	19	17.8	9.6	116.38	111.66	60	51	27	16	0.55	0.69
8	F	62	None	PBC	26	22.1	9.4	118.20	123.28	53	50	18	18	0.66	0.64
9	M	44	None	HBV + HCC	14	15.4	10.6	111.30	108.41	56	64	23	28	0.59	0.56

As in Table 1.

There were 13 patients with PFV <85 ml/min/100 g (ligation group) and 9 with PFV ≥85 ml/min/100 g (non-ligation group). As shown in Table 3, recipients with ligation group and non-ligation group were similar in age, end-stage liver disease (MELD) score, the portal vein diameter (PVD), and the incidence of portal vein thrombosis (PVT). Donors to patients in the two recipient groups showed no differences in liver graft weight and cold ischemia time (Table 3). Liver grafts with mild-moderate steatosis were similar in the 2 groups, and grafts with severe steatosis did not included in the 2 groups. Only 1 liver graft was found severe IRI with centrocentrilobular necrosis, and this graft was in ligation group. However, SRS diameter was significantly larger in the ligation group than that in the non-ligation group (Table 3).

**Table 3:** The characteristics of recipient and donor in 2 groups.

Characteristics	Ligation (*n *= 13)	Non-ligation (*n *= 9)	*p* value
Age (year)	48.15 ± 2.86	53.56 ± 3.85	0.1759
Male, *n* (%)	8 (61.54%)	6 (66.67%)	0.8058
MELD	23.15 ± 1.50	22.00 ± 2.04	0.6599
PVT, *n* (%)	7 (53.85%)	4 (44.44%)	0.6646
PVD (mm)	9.85 ± 0.30	9.67 ± 0.30	0.6755
Liver graft weight (g)	1462.0 ± 57.5	1490.0 ± 58.3	0.7399
Cold ischemic time (h)	7.92 ± 0.59	7.39 ± 0.54	0.5314
SRS diameter (mm)	22.92 ± 4.18	16.24 ± 3.60	0.0009

As in Table 1.

PFV was elevated in all cases with SRS occlusion and increased significantly from 68.74 ± 8.77 to 116.80 ± 16.50 ml/min/100 g (*p *< 0.0001) after ligation (Table 1 and Figure 4). On day 7 after transplantation, PFV was decreased compared to immediately after SRS ligation, although the difference was not statistically significant (Table 1). The PSV of the HA decreased immediately after SRS ligation from 58.17 ± 14.87 to 46.67 ± 13.28 cm/s (*p *= 0.0013), with no significant difference in PSV immediately after ligation as compared to day 7 post transplantation (Table 1). In contrast, SRS clamping had no effect on EDV and RI during LT (Table 1). As shown in Table 2, there was no difference between the intraoperative PFV and the PFV on day 7 post transplantation (107.90 ± 14.86 vs. 113.50 ± 12.93 ml/min/100 g; *p *= 0.3173) in the non-ligation group.

One patient had a large spontaneous GRS, and the recipient gastric coronary vein was end-to-end anastomosed with the graft PV; after clamping the GRS, PFV increased from 77.95 to 126.03 ml/min/100 g (Figure 5).

The median follow-up was 31.36 months (range: 10–54 months); at the time of writing, no HA flow-related biliary complications or SRS ligation-related kidney injury occurred in 13 patients, 21 of the 22 patients were alive with no evidence of PV complications. One patient (ligation group) died of severe infection during the perioperative period but this was unrelated to the procedure of SRS ligation.

## Discussion

Spontaneous portosystemic shunts such as SRS are portocaval communications in patients with chronic liver disease and portal hypertension (15). A large SRS was observed in 3.05% of liver transplant recipients in our study, although a much higher incidence has been reported in patients with chronic liver disease (16). In the present study, the diameter of SRS was larger in the ligation group than that in the non-ligation group, suggesting that SRS diameter is associated with PFV reduction during LT. PFV increased nearly 2 folds when the shunt was occluded by SRS clamping, an effect that persisted to the day 7 post transplantation. When the SRS was clamped, the increase in PV flow was followed by a reduction in the PSV of the HA, consistent with the previously reported hepatic arterial buffer response to PV flow (17).

In the present study, no SRS ligation-related kidney injury or HA flow-related biliary complications were occurred in 13 patients. For patients without PVT or with resectable PVT, LRVL is the recommended surgical treatment for SRS in LT, however, previous studies have shown that ligation of the left RV can lead to sustained and elevated serum creatinine levels and decreased kidney size in recipients (12, 18). RPA, in which the donor PV is anastomosed end-to-end with the left renal vein, is another effective treatment for SRS, especially in patients with PVT. However, the procedure is complicated and may require an additional interposed vein graft to connect the left RV to the PV. Additionally, because PV flow is derived from both a large SRS and the left renal vein, portal hyperperfusion can occur during living donor LT (PFV >250 ml/100 g/min) (11, 19). A recent study reported a 31.3% (5/16) incidence of upper gastrointestinal bleeding and 43.8% (7/16) incidence of postoperative ascites when portal hypertension was not fully relieved after RPA (20). In our patients, direct ligation of a large SRS was performed safely and with satisfactory PV flow and no instance of either portal hypoperfusion or PV hyperinflow.

The hepatic arterial buffer response is an intrinsic mechanism of the liver to maintain total hepatic blood flow when portal perfusion decreases (21). Adenosine is a potent vasodilator that acts directly on the HA but not the PV; it is secreted at a constant rate and is washed away by PV flow. A reduction in PV flow leads to accumulation of adenosine and dilation of the HA (22). The occurrence and degree of the arterial buffer response should be considered when deciding whether the SRS should be ligated, as a significant reduction in HA flow can increase the risk of biliary complications during LT (23).

In all of our patients, the SRS was located below the distal pancreas and beside the inferior mesenteric vein, it was easily identified by locating and following the LRV. The retroperitoneal tissue was relatively loose, and the SRS was always free without extra branches, thus, the large SRS was not technically difficult to identify, nor was it dangerous to isolate. In the present study, no procedure-related complications occurred in patients who underwent direct SRS ligation. However, a patent PV is mandatory for direct ligation of the SRS. Additionally, SRS ligation should not be attempted in patients with unresectable PVT or in whom angioplasty of PV stenosis has failed.

There were several limitations to the study. Firstly, it was designed as a retrospective analysis of a relatively small number of patients. Secondly, the judgment of portal vein blood flow by Doppler US is not very precise, while measuring PFV by ultrasonic flowmetry is more objective, this was not always possible at our center. Thirdly, the PFV reference value of 85 ml/min/100 g of our center was calculated from a historical non-SRS cohort of 100 deceased adult liver transplant donors. However, a previous study used PFV <1200 ml/min after reconstruction as a reference value to determine whether the SRS was disconnected by LRVL (17). Prospective studies with a larger sample size are needed to establish more reasonable reference values.

In conclusion, we demonstrated that direct ligation of a large SRS was effective in achieving nearly 2 folds portal inflow. The large SRS was easily identified and isolated, and no SRS ligation-related complications injury were found in the ligation group. Additionally, the surgical procedure for SRS ligation is in line with normal physiology and minimally invasive. Direct ligation of large SRS could be an effective and safe surgical procedure to overcome the problem of portal hypoperfusion during LT.

## Data Availability

The original contributions presented in the study are included in the article/Supplementary Material, further inquiries can be directed to the corresponding author/s.
